# Test-retest reliability and sensitivity of the 20-meter walk test among patients with knee osteoarthritis

**DOI:** 10.1186/1471-2474-14-166

**Published:** 2013-05-10

**Authors:** Jillian M Motyl, Jeffrey B Driban, Erica McAdams, Lori Lyn Price, Timothy E McAlindon

**Affiliations:** 1 Division of Rheumatology, Tufts Medical Center, 800 Washington Street, Box #406, Boston, MA 02111, USA; 2 The Institute for Clinical Research and Health Policy Studies, Tufts Medical Center, and Tufts Clinical and Translational Science Institute, Tufts University, 800 Washington Street, Box #63, Boston, MA 02111, USA

**Keywords:** Knee, Osteoarthritis, Functional assessment

## Abstract

**Background:**

The 20-meter walk test is a physical function measure commonly used in clinical research studies and rehabilitation clinics to measure gait speed and monitor changes in patients’ physical function over time. Unfortunately, the reliability and sensitivity of this walk test are not well defined and, therefore, limit our ability to evaluate real changes in gait speed not attributable to normal variability. The aim of this study was to assess the test-restest reliability and sensitivity of the 20-meter walk test, at a self-selected pace, among patients with mild to moderate knee osteoarthritis (OA) and to suggest a standardized protocol for future test administration.

**Methods:**

This was a measurement reliability study. Fifteen consecutive people enrolled in a randomized-controlled trial of intra-articular corticosteroid injections for knee OA participated in this study. All participants completed 4 trials on 2 separate days, 7 to 21 days apart (8 trials total). Each day was divided into 2 sessions, which each involved 2 walking trials. We compared walk times between trials with Wilcoxon signed-rank tests. Similar analyses compared average walk times between sessions. To confirm these analyses, we also calculated Spearman correlation coefficients to assess the relationship between sessions. Finally, smallest detectable differences (SDD) were calculated to estimate the sensitivity of the 20-meter walk test.

**Results:**

Wilcoxon signed-rank tests between trials within the same session demonstrated that trials in session 1 were significantly different and in the subsequent 3 sessions, the median differences between trials were not significantly different. Therefore, the first session of each day was considered a practice session, and the SDD between the second session of each day were calculated. SDD was −1.59 seconds (walking slower) and 0.15 seconds (walking faster).

**Conclusions:**

Practice trials and a standardized protocol should be used in administration of the 20-meter walk test. Changes in walk time between −1.59 seconds (walking slower) and 0.15 seconds (walking faster) should be considered within the range of normal variability of 20-meter walking speed. The primary limitation of our study was a small sample size, which may influence the generalizability of our findings.

## Background

Osteoarthritis (OA) is a major debilitating disease affecting more than 27 million individuals in the United States [1], with numbers expected to rise as the obesity epidemic continues to increase [2]. Knee OA is amongst the most common and functionally limiting types of OA, causing pain and functional impairment (e.g., slower gait speed) [3,4]. In both clinical studies and rehabilitation clinics, it is vital to have assessments that can monitor patients’ physical function over time.

Gait speed is an important functional outcome among patients with knee OA. Physical therapy [5],exercise programs [6] and drug interventions [7] may improve gait speed and can be easily assessed using 10-, 20-, or 50-meter walk tests [8-14]. Unfortunately, each of these short-distance walk tests may have different sensitivities. The 20-meter walk test is frequently used in clinical trials and cohort studies involving individuals with OA, as well as in physical therapy [15-22]. While a recent systematic review [23] determined the 40-meter and 50-ft walk tests have the best measurement evidence, this review did not include the 20-meter walk test. Due to the 20-meter walk test’s frequent use in OA studies, it is important to determine its sensitivity and test-retest reliability using a symptomatic population with mild to moderate OA, a population often used in clinical trials for OA. There is also a limited amount of literature regarding gait speed changes over short periods of time (less than 30 days). Research on changes in gait speed over short periods of time, when no changes should have occurred, is important in understanding what kind of change is attributed to real change in gait speed versus a patient’s normal variability. Smallest detectable differences (SDD) would allow both clinicians and researchers to determine if a patient’s gait speed is worsening or improving based on 20-meter walk time. Therefore, the purpose of this study was to determine the test-retest reliability and sensitivity of the 20-meter walk test at a self-selected pace among patients with mild to moderate knee OA and to suggest a standardized protocol for future 20-meter walk test administration in both rehabilitation and research settings.

## Methods

This study is reported according to the Guidelines for Reporting Reliability and Agreement Studies (GRRAS) [24].

### Participants

The study sample included 15 consecutive participants with mild to moderate knee OA attending screening (Day 1) and baseline (Day 2) visits between July 2011 and December 2011 for a randomized-controlled trial of intra-articular corticosteroid injections for knee OA (NIH RO1AR057802-01, NCT01230424). All participants met the American College of Rheumatology clinical criteria for OA; had radiographic knee OA, as defined by Kellgren-Lawrence grade 2 or 3 [25]; and knee synovitis, defined by a synovial pouch depth greater than 2.0 mm measured by ultrasound [26,27]. All participants were also required to have knee pain symptoms, defined as a WOMAC Osteoarthritis Index (version 3.1, 5-point Likert) pain subscore ≥ 2 at the beginning of the Day 1 visit. Symptoms were reassessed at the beginning of the Day 2 visit, but this did not influence eligibility. Participants were instructed to withhold use of any non-steroidal anti-inflammatory medications or analgesics within 48 hours of their study visits. There were no other restrictions imposed. The Tufts University/Tufts Medical Center Institutional Review Board approved this ancillary study, and informed consent was collected prior to data collection.

### 20-Meter walk (self-selected pace)

**Figure 1:**
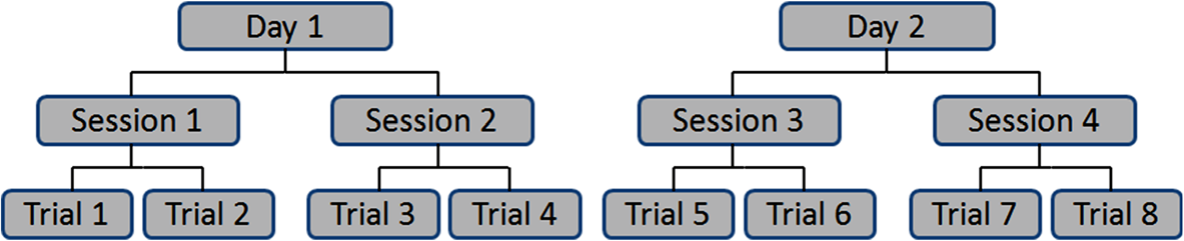
Walk test administration trials.

The 20-meter walk test was performed at two visits 8 to 20 days apart. Participants completed four trials of the 20-meter walk test at each visit, which were equally divided into two sessions (see Figure 1). Sessions were approximately ten minutes apart. Participants completed eight trials during four sessions. The primary investigator (JM) administered all walk tests using a standardized script and protocol (see Appendix). All walk tests were conducted at a self-selected pace.

Participants completed the 20-meter walk test in a 40-meter long, unobstructed hallway. The primary investigator measured the 20-meter walk course with a Redi-measure wheel (American Marking Corp, Omaha, Nebraska) in the forward and reverse directions to ensure accuracy. Two 20 cm long strips of bright orange tape were placed at the beginning and end of the course.

All participants wore comfortable, soft-soled shoes and did not use walking aids. The primary investigator demonstrated the walk test at a relaxed pace, exaggerating the instruction to pass the orange line by 3 to 4 steps. The assessor then instructed the participants to place their toes at the edge of the tape marking the beginning of the course. Participants were prompted, “When I want you to start, I will say ready, begin. Ready? Begin!” When the participant began walking, the stopwatch was started, and the assessor followed slightly behind and to the right of the participant. The assessor stopped timing as soon as the participant’s first heel completely crossed the strip of orange tape. The assessor did not stop the time until the participant’s entire foot completely crossed the far edge of the tape. Participants had a 15 to 30 second break between trials.

If the participant began to walk at a pace that was obviously not their normal self-selected walking speed as determined by the assessor (e.g., running or jogging), the test was immediately stopped. Participants were prompted with a script to walk at a comfortable pace and told to begin again (see Appendix).

### Statistical analyses

Descriptive characteristics were calculated for participant characteristics and walk times. Since previous research typically only performs one or two walking trials, we calculated the differences between walk times (raw and average) as initial walk time minus follow-up walk time. Therefore, negative differences represent a participant who was slower during the follow-up observation and positive differences characterize a participant with a faster walking time during the follow-up observation.

#### Test-retest reliability between trials

We assessed differences between trials within each of the four sessions to determine if trials were different within a session. Walk times and differences between trials were not normally distributed, therefore, Wilcoxon signed-rank tests were performed to assess the differences between 2 trials within session (significance was defined as p ≤ 0.01 to account for multiple comparisons). With a sample size of 15 participants the Wilcoxon signed-rank tests could detect a large difference (standardized effect size [d] = 1.00) between trials based on an alpha level of 0.01 and power of 0.80 (calculated with G*Power 3.1.2). Spearman correlations were performed to assess the relationship between the 20-meter walk times between trials within each session.

#### Test-retest reliability between sessions

Five Wilcoxon signed-rank tests were performed to assess the differences of the average 20-meter walk times between session 1 and the 3 subsequent sessions as well as session 2 compared to the 2 subsequent sessions (significance was defined as p ≤ 0.01 to account for multiple comparisons). With a sample size of 15 participants the Wilcoxon signed-rank tests could detect large differences (standardized effect size [d] = 1.00) between sessions based on an alpha level of 0.01 and power of 0.80 (calculated with G*Power 3.1.2). Spearman correlations were performed to assess the relationship between the average 20-meter walk times across trials. Confidence intervals for the Spearman correlations were calculated with PASS 11.0.10 (NCSS, LLC, Kaysville, Utah).

#### Sensitivity: smallest detectable differences (SDD)

Data from the second sessions of both days (sessions 2 and 4) were used to determine the SDD in average 20-meter walk times. The second session from each day was selected because our data indicated that the first session may not be valid and, therefore, should represent practice trials. Since differences between session 2 and 4 were not normally distributed we calculated SDD based on the median difference between sessions. To calculate a more precise estimate of the SDD, we conducted 200 bootstrapped samples and determined the median of each sample. We reported the SDD as the 2.5% and 97.5% percentiles from the empirical distribution of the median from the boostrapped samples. All analyses were performed in SAS 9.2 (Cary, NC), except when noted otherwise.

## Results

**Figure 2:**
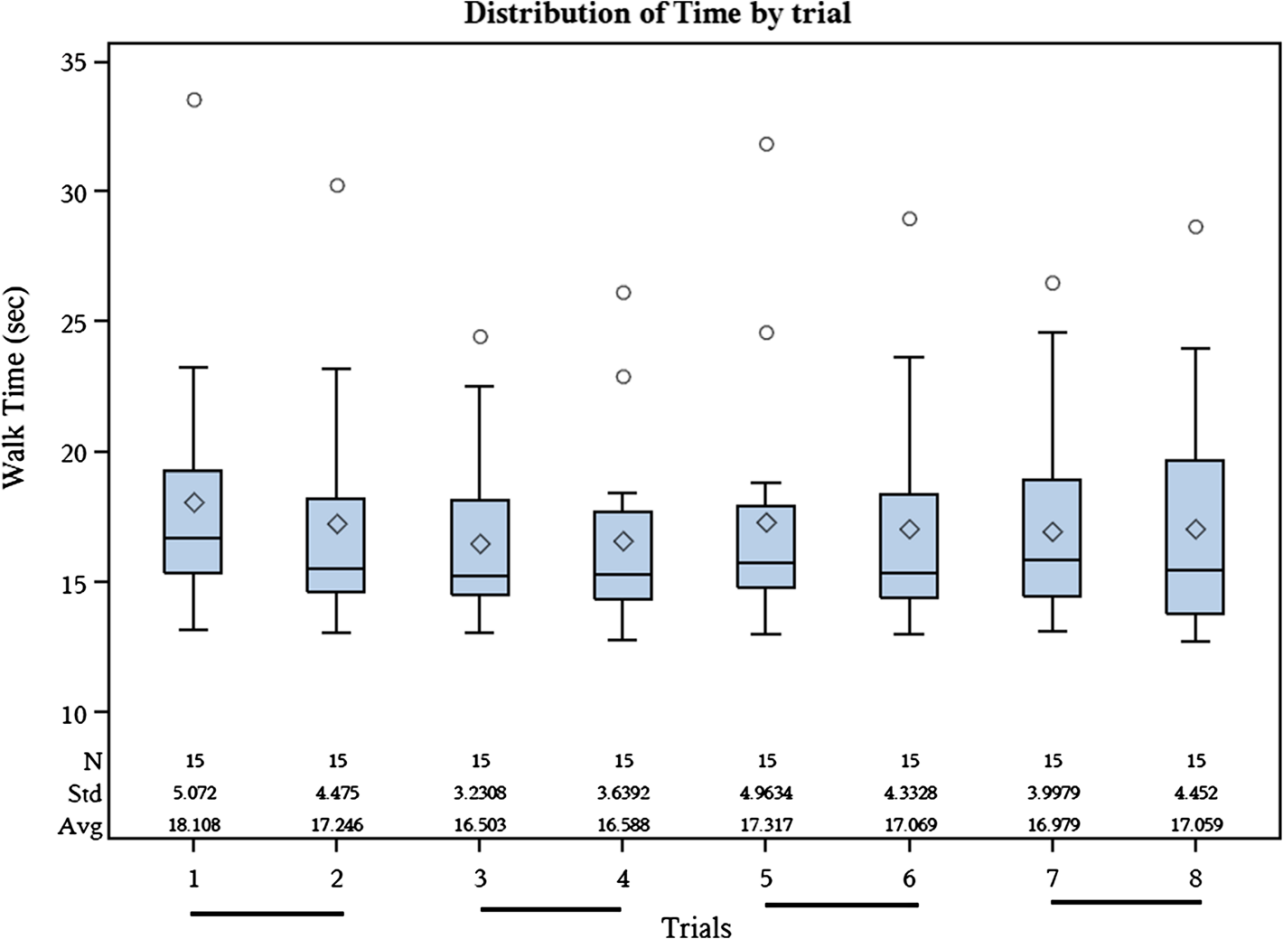
**Distribution of 20 meter walk times (seconds) by trial (whiskers = interquartile range ± 1.5 times the interquartile range, boxes = interquartile ranges, diamond = mean values, horizontal line in each box = median values).** Lines under each set of trials indicate trials performed during the same session.

Fifteen participants volunteered to perform 2 walking trials without assistive devices during each of the 4 sessions (average time between visit days = 13.9 days; range = 8 to 20 days), with 8 walking trials total. Participant characteristics are described in Table 1. Patient-reported symptoms, based on total WOMAC pain score, were not significantly different between day 1 and day 2 (t(14) = 1.70, *p* = 0.11). Between days, 5 participants had no change in WOMAC pain score, 7 changed WOMAC pain scores by 1 unit, and 3 participants changed WOMAC pain score by 2 units. The distribution of 20-meter walk times by trial is in Figure 2.

**Table 1:** Participant characteristics (n = 15)

**Variable**	**Distribution**
Female (%, n)	53% (8)
Caucasian (%, n)	67% (10)
Age (years; mean (sd))	61.0 (7.8)
Body mass index (kg/m^2^; mean (sd))	28.9 (5.4)
Kellgren-Lawrence Grade = 2 (%, n)	20% (3)
Kellgren-Lawrence Grade = 3 (%, n)	80% (12)
Day 1 WOMAC Pain Score (mean (sd))	5.3 (1.3)
Day 2 WOMAC Pain Score (mean (sd))	4.9 (2.0)

### Test-retest reliability between trials

Wilcoxon signed-rank tests between trials within the same session demonstrated that trials in session 1 were significantly different (p < 0.01) with a median difference of 0.78 seconds (− 0.06 m/s; Table 2). In the subsequent 3 sessions, the median differences between trials ranged from −0.15 to 0.13 seconds (−0.01 to 0.02 m/s) and were not significantly different (Table 2). Within sessions, trials had good correlations (r ≥ 0.90).

**Table 2:** Within-session comparisons and associations between trials

	**1st Trial**	**2nd Trial**	**Median difference**		**Spearman**	**Average of 2 trials**
	**Median (25%, 75%)**	**Median (25%, 75%)**	**(sec)**	**p value**	**Correlation**	**Median (25%, 75%)**
	**(sec)**	**(sec)**			**(95% CI)**	**(sec)**
Day 1						
Session 1	16.72 (15.38, 19.28)	15.53 (14.60, 18.18)	0.78	< 0.01	0.94 (0.83, 0.98)	16.11 (14.99, 19.07)
Session 2	15.28 (14.47, 18.12)	15.31 (14.32, 17.65)	−0.15	0.63	0.90 (0.72, 0.97)	15.17 (14.85, 17.97)
Day 2						
Session 3	15.71 (14.80, 17.94)	15.35 (14.37, 18.37)	0.05	0.55	0.97 (0.91, 0.99)	15.53 (14.78, 18.16)
Session 4	15.80 (14.41, 18.90)	15.44 (13.75, 19.66)	0.13	0.98	0.98 (0.94, 0.99)	15.62 (14.16, 19.44)

Note. P values are based on Wilcoxon signed-rank tests. 95% CI = 95% confidence interval for Spearman Correlation Coefficient.

### Test-retest reliability between sessions

Differences and associations between sessions were evaluated based on the average of two trials (Table 3). Based on Wilcoxon signed-rank tests, walking times in session 1 were slower than session 2 (median difference = 0.53 seconds, -0.04 m/s; Table 3). No other sessions were significantly different (median differences ranging from −0.14 to 0.31 seconds, -0.03 to 0.01 m/s); however, we detected a trend that participants walked slower in session 3 compared to session 2 (p = 0.07). Furthermore, we detected very strong associations between session 2 and session 3 or 4 (Spearman r = 0.94 and 0.95). In contrast, session 1 did not relate as well with sessions performed on the second day (session 3 and 4; r = 0.78; p ≤ 0.01; for both sessions after rounding).

**Table 3:** Between-session comparisons and associations

	**Median difference**		**95% CI of the**	**Spearman correlation**
			Median*	**(95% CI)**
**Comparison**	**(sec)**	**p value**	**(sec)**	
Session 1 – Session2	0.53	< 0.01	0.15, 0.91	0.88 (0.67, 0.96)
Session 1 – Session 3	0.31	0.22	−0.41, 0.71	0.78 (0.45, 0.92)
Session 1 – Session 4	0.12	0.28	−0.76, 1.33	0.78 (0.45, 0.92)
Session 2 – Session 3	−0.14	0.07	−1.13, 0.07	0.95 (0.85, 0.98)
Session 2 – Session 4	−0.04	0.19	−1.59, 0.15	0.94 (0.83, 0.98)

Note. P values are based on Wilcoxon signed-rank tests. 95% CI = 95% confidence interval. *Based on bootstrapping sample.

### Sensitivity: smallest detectable difference

Smallest detectable differences in average 20-meter walk times were based on differences between session 2 and session 4 (the second sessions of each day). The median difference between sessions was −0.04 seconds (0.00 m/s). Based on the empiric 95% confidence interval of the bootstrapped medians, the SDD was change less than −1.59 seconds (> 0.07 m/s; walking slower) or change greater than 0.15 seconds (< −0.03 m/s; walking faster). Based on this SDD, two participants walked slower in the final session (Participant 1: average time = 18.44 seconds, difference = −1.99 seconds; Participant 2: average time = 16.45 seconds, difference = −1.75 seconds) and one participant walked faster (average time = 14.66 seconds, difference = 0.62 seconds). There was a potential systematic bias of participants walking slower during the first and second sessions on day 2 compared to the second session of day 1 (10 [67%] participants walked slower in session 4 compared to session 2; based on differences below zero).

## Discussion

Using a standardized protocol and one investigator to eliminate variability introduced among raters, we found a significant difference between the trials in session 1 and the average times of session 1 and session 2. We detected a trend that participants walked slower during session 3 compared to session 2. Our findings may indicate that practice trials should be included when performing the 20-meter walk test. Greater session 1 and 3 walking times as well as discrepancies between trials in the first session may be due in part to a learning effect and by participants acclimating to the test. Furthermore, the lack of correlation between session 1 and the following sessions calls into question the test-retest reliability of the first session and thus the validity of walking times from that session. Based on these findings we propose that clinicians who use the 20-meter walk as a determinate of physical function change over time should conduct practice trials, average the results of two trials, and consider the potential for a systematic bias at follow-up (i.e., walking slower).

Practice walks have been found to increase the test-retest reliability of the 400-meter walk [28], but have not been explored for the shorter walk tests. Despite the numerous studies using the 20-meter walk as a physical function outcome, none to our knowledge have reported using practice trials. Participant acclimation to the test may be vital to collecting a valid walk time [29,30], but, to our knowledge, this has not been explicitly studied for the 20-meter walk test. Session 3, despite being the first session of day 2, was not significantly different from the other sessions; however, there was a trend indicating that participants walked slower in session 3 compared to session 2. This suggests that to be cautious we should always consider using practice trials before administering the walk tests.

Based on the SDD, we determined that change in 20-meter walk times between −1.59 seconds (walking slower) and 0.15 seconds (walking faster) should be considered within the normal variation for the 20-meter walk test at self-selected pace. Standardization of SDD among researchers and clinicians will improve reporting and interpretation of change outside of normal variability for patients with knee OA. While the SDD describes the range of normal variation it does not represent the amount of change that may be clinically meaningful. Therefore, future research may be warranted to determine how much change in the 20-meter walk test at a self-selected pace may be clinically meaningful to a patient (e.g., associated with self-reported improvement or worsening of function).

While SDD is often considered to be evenly distributed around zero change it is important to note that we identified a systematic bias that indicated participants are more likely to walk slower in the final session than the second session of day 2. If, however, we used the first session from day 1, and did not include practice trials, we would find an opposite bias (people walked faster in subsequent session). This finding further highlights the potential importance of practice trials when evaluating 20-meter walk times. These potential biases should be considered when evaluating if the 20-meter walk time improved or worsened. Supporting the current SDD were our findings that the three participants with differences in walk times outside of the SDD were also those who had changes in symptoms, therefore their variation in walk time may be real change.

While this study offers important insight into the 20-meter walk test, one limitation of this study is that the data may not be generalizable to other walking distances that are occasionally performed (e.g., 6-minute walk test). Another important limitation of this study may be our small sample size. This may influence the generalizability of these findings; however, they were representative of patients with mild to moderate knee OA that participate in clinical trials in age and gender [31,32]. This is beneficial since the 20-meter walk has a high test-re-test reliability among patients with end-stage OA awaiting knee replacements [33]. Our study along with previous research may indicate that the 20-meter walk test is a robust assessment of gait speed among patients with knee OA. Furthermore, our sample size was similar to other reliability studies [34-37] and was sufficient to demonstrate that trial 1 and 2 in the first session were statistically different and that the correlations between the average walk time in session 1 and the other trials were smaller than the correlations between session 2 and the subsequent sessions.

Another limitation of our study sample was that it included only a few participants with slower walking times, allowing for only broad generalizations regarding potential limitations of the validity of this measure for slower walkers (i.e., patients with greater functional impairments). The slowest walkers had the biggest differences in walking times from Day 1 to Day 2. Future research should further explore the reliability and sensitivity of the 20-meter walk test among slower walkers. Furthermore, it may be beneficial for future research to explore potential sources of variation in walking times (e.g., walking speed, assessor, time between visits, time of assessment during the day) and how much they contribute to variation in walking times.

## Conclusions

In summary, our study demonstrated the first two walking trials may not represent a participant’s normal self-paced walking speed and lacked good test-retest reliability. Therefore, practice trials are advised prior to a valid measure of a participant’s walk time and gait speed. We also advocate that changes in 20-meter walk times at a self-selected pace between −1.59 seconds (walking slower) and 0.15 seconds (walking faster) may represent normal variability in walking speed among those with mild to moderate knee OA.

## **Appendix: 20-Meter Walk Test Protocol**

### **Equipment:**

•Stopwatch

•Bright colored tape

•25 meter long hallway

•Redi-measure wheel (in meters)

### **Setting up the course:**

•Pick a stretch of hallway at least 25 meters in length. Ideally, this area would not be highly travelled.

•Place one piece of tape on the ground. This will be the starting line.

•Using the Redi-measure wheel, measure out a 20-meter course down the hallway.

•Mark the other end with another piece of tape; this will be the finish line.

•To ensure accuracy, re-measure the course in the reverse direction.

### **Performing the test:**

•The participant should be wearing comfortable, soft soled shoes for this test.

•If the participant reports any significant discomfort, or does not agree to participate in the explained test, they should not be forced.

•Participants may use a cane or other walking aid to walk the 20-meter course if they feel it is necessary. However, keep in mind that many participants only use their walking aids while outside of the home, so do not assume they will need it just because they brought an aid to the clinic.

•Read the participant the following script:

### **Script 1**

“Now I am going to observe how you normally walk. If you use a cane or other walking aid, and you feel you need it to walk a short distance, then you may use it. This is our walking course. I want you to walk to the other end of the course, at your usual speed, just as if you were to walk down the street to go to the store. Walk 3 – 4 steps past the other end of the tape before you stop. I will walk slightly behind you. I will now demonstrate the walk to you”

•Demonstrate the walk. Do not walk at your normal pace, rather, walk at a relaxed, leisurely pace all the way past the other end of the course. Exaggerate how far to walk by taking at least 3 or 4 steps past the finish line.

•Return back at your normal speed.

•Ask the participant if they feel safe performing the test, if so, assist them to the starting line.

•Be sure to get their toes as close as possible to the edge of the course.

•The examiner should only have a stopwatch. Read the following to the participant:

“When I want you to start, I will say ready, begin. Ready? Begin!”

•Press the start/stop button on the stopwatch as soon as the participant starts to move across the starting line.

•Follow the participant all the way down to the other end of the course. The examiner should be postioned slightly behind and to the side of the participant, keeping a close eye on the participant’s feet.

•The test is over when the participants first foot completely crosses the line. If the participant’s foot lands on the line, do not stop the timer until one heel completely crosses the finish line.

•Inform the participant that you are to go to the other end of the course and record the walking time. If they require a chair, provide one.

•NOTE: If the participant begins the test and is obviously not walking at their normal pace (e.g. running or jogging please read the following to the participant: *“Please stop the test. Is this how you normally walk down the street going to the store? If so we will begin again. If not, please remember to walk at your normal walking pace.”* and then have participant start the test again.

•After recording the walk information, read the following script to the participant:

### **Repeating the Walk Test**

“Now I want you to repeat the walk. Remember to walk at your usual pace and walk all the way past the other end of the course before you stop. I will be walking with you.”

•If the participant feels safe performing the test, assist them to the starting line.

•Be sure to get their toes as close as possible to the edge of the course.

•The examiner should only have a stopwatch.

•Read the following to the participant:

“When I want you to start, I will say ‘Ready? Begin!’ Ready? Begin!”

•Press the start/stop button on the stopwatch as soon as the participant starts to move across the starting line.

•Follow the participant all the way down to the other end of the course. The examiner should be positioned slightly behind and to the side of the participant, keeping a close eye on the participant’s feet.

•The test is over when the participant’s first foot completely crosses the line. If the participant’s foot lands on the line, do not stop the timer until one heel completely crosses the finish line.

Repeat the walk test two more times. Assist the participant to their seat and record all information.

## Abbreviations

OA: Osteoarthritis; SDD: Smallest detectable difference; WOMAC: Western Ontario and McMaster Universities Osteoarthritis Index.

## Competing interests

The authors declare that they have no competing interest.

## Authors’ contributions

JM contributed to the conception and design, acquisition of data, analysis and interpretation of data, drafting/revisions of article, as well as final approval of the article. JBD contributed to the conception and design, analysis and interpretation of data, drafting/revisions of article, as well as final approval of the article. EM contributed to the conception and design, drafting/revisions of article, as well as final approval of the article. LLP contributed to the contributed to the drafting/revisions of article and the final approval of the article. TEM contributed to the conception and design, analysis and interpretation of data, drafting/revisions of article, as well as final approval of the article. All authors read and approved the final manuscript.

## Pre-publication history

The pre-publication history for this paper can be accessed here:

http://www.biomedcentral.com/1471-2474/14/166/prepub
